# Ploidy dynamics and evolvability in fungi

**DOI:** 10.1098/rstb.2015.0461

**Published:** 2016-12-05

**Authors:** Noa Blutraich Wertheimer, Neil Stone, Judith Berman

**Affiliations:** 1 Department of Molecular Microbiology and Biotechnology, Tel Aviv University, Britannia 418, Ramat Aviv, Israel; 2 Institute of Infection and Immunity, St George's, University of London, London SW17 0RE, UK

**Keywords:** drug resistance, aneuploidy, loss of heterozygosity, trimeras, adaptation

## Abstract

Rapid responses to acute stresses are essential for stress survival and are critical to the ability of fungal pathogens to adapt to new environments or hosts. The rapid emergence of drug resistance is used as a model for how fungi adapt and survive stress conditions that inhibit the growth of progenitor cells. Aneuploidy and loss of heterozygosity (LOH), which are large-scale genome shifts involving whole chromosomes or chromosome arms, occur at higher frequency than point mutations and have the potential to mediate stress survival. Furthermore, the stress of exposure to an antifungal drug can induce elevated levels of LOH and can promote the formation of aneuploids. This occurs via mitotic defects that first produce tetraploid progeny with extra spindles, followed by chromosome mis-segregation. Thus, drug exposure induces elevated levels of aneuploidy, which can alter the copy number of genes that improve survival in a given stress or drug. Selection then acts to increase the proportion of adaptive aneuploids in the population. Because aneuploidy is a common property of many pathogenic fungi, including those posing emerging threats to plants, animals and humans, we propose that aneuploid formation and LOH often accompanying it contribute to the rapid generation of diversity that can facilitate the emergence of fungal pathogens to new environmental niches and/or new hosts, as well as promote antifungal drug resistance that makes emerging fungal infections ever more difficult to contain.

This article is part of the themed issue ‘Tackling emerging fungal threats to animal health, food security and ecosystem resilience’.

## 1. Introduction

Emerging fungal infections continue to pose significant threats to animals, plants and humans, causing disease outbreaks and even posing a threat to food security [1]. The emergence of new fungal threats appears to require the rapid adaptation of fungal pathogens to changes in environmental conditions. Some of these environmental changes, for example changes in ambient temperatures or levels of humidity, make host animals and plants more vulnerable to fungal infection (reviewed in [1,2]). In addition, fungi appear able to rapidly adapt in a manner that involves complex and heterogeneous genome dynamics. These often result in a high level of aneuploidy and loss of heterozygosity (LOH) [3], which can lead to clinical consequences such as changes in virulence or response to antifungal drugs [4]. This genomic diversity may contribute to the emergence of new fungal threats by providing a selective advantage in certain conditions.

Understanding the mechanisms underpinning large and rapid genome changes that affect virulence and other fungal properties requires investigation at the molecular, genetic and cell biological level coupled with experimental evolution of known isolates both *in vitro* and *in vivo.* Recent work suggests that aneuploidy is a common property of pathogenic and environmental isolates of unicellular fungi, ranging from basidiomycetes such as *Cryptococcus neoformans*, to ascomycete yeasts, including wild isolates of baker's yeast *Saccharomyces cerevisiae* and *Candida albicans*, a common commensal of humans and the most prevalent cause of fungal infections of humans [1,5–9].

The appearance of drug resistance in a population of susceptible cells can only be understood, as Theodosius Dobzhansky argued, ‘…in the light of evolution’ [10]. In most cases, microbes that evolve resistance must cope with exposure to drug concentrations well above their ‘minimal inhibitory concentration’ (MIC). The overriding strategy for long-term survival and adaptation is to alter either genome sequence and/or genome organization. This is especially true for fungi and other eukaryotes that, unlike bacteria, do not participate in active, high-frequency lateral gene transfer. Accordingly, changes to the existing genome must appear rapidly and must provide some selective benefit in the presence of the drug. This first rapid adaptation step may be suboptimal, allowing the organism either to avoid or to neutralize the stress. Critically, it must facilitate the subsequent acquisition of additional mutations that provide more refined solutions [11].

Fitness, a key factor in the emergence of new fungal threats, is a relative feature of genotype, phenotype, environmental conditions and the fitness of other organisms occupying the same environment. A useful concept for considering population dynamics, which are critical for understanding the emergence of new variants, is the fitness landscape. First proposed by Sewall Wright as the multidimensional distribution of possible genotypes as a function of their fitness [12], fitness landscapes have been described as a function of genotype or phenotype [13] and also as a function of the environmental challenges facing an organism [14]. We assume that emerging fungal pathogens have attained a higher position in the landscape because of changes in either their genotype/phenotype or environmental factors, including the host niche and the presence of microbial competitors [15].

With respect to genotypes and resulting phenotypes, wild isolates of pathogens often differ from each other and from laboratory strains at tens of thousands of single nucleotide polymorphisms (SNPs) that potentially modify phenotypes via a myriad of mechanisms. Similarly, different pathogenic isolates exhibit different levels of resistance and tolerance to drugs. By contrast, laboratory studies often use refined strains that have been engineered to facilitate experimental procedures. For example, laboratory strains of the model yeast *S. cerevisiae* carry mutations in *FLO8,* which activates other genes that promote cell aggregation and clumping, a phenotype critical for life in the ‘wild’ and inconvenient for laboratory work [16]. Importantly, wild and laboratory strains may exhibit different responses to genome changes such as aneuploidy [17]. Thus, for laboratory experiments to be generalized to the situation in the patient or the field, it is important to ask if the phenotypes measured can be observed in a range of strain backgrounds.

In terms of environmental conditions, laboratory growth conditions strive to optimize rapid log phase growth and the attainment of high biomass levels. Organisms adapted to these laboratory conditions occupy a high**-**altitude position in a landscape. In contrast, addition of an antimicrobial drug to which a population is susceptible will shift the population to a lower position on the landscape. *In vivo*, fungal pathogens usually experience nutrient limitations, which likely reflect an intermediate position on the fitness landscape. Even within a single host, environmental niches for fungal growth can vary considerably in temperature, pH, oxygen concentration and nutrient availability. Furthermore, these conditions can fluctuate (e.g. diurnal and seasonal temperature shifts; oxidative stress from immune cell invasion) and cells can transit from one extreme niche to another (e.g. pH and oxygen availability levels from the oral cavity to the stomach and colon). It appears that organisms have evolved mechanisms facilitating adaptation to these routine environmental shifts [18]. By contrast, abrupt and severe environmental shifts, such as sudden exposure to an antifungal drug, may demand different adaptive strategies.

This review focuses on the role of aneuploidy, whole genome ploidy and LOH in the rapid emergence of new phenotypes. Aneuploidy is a karyotypic state in which chromosome copy numbers are not balanced. For normally diploid organisms, this can include monosomy (one copy), trisomy (three copies) or even higher numbers (e.g. tetrasomy (four copies of a single chromosome)) of one or more different chromosomes. Aneuploidy is often owing to chromosome mis-segregation; it also can arise via reduplication of a whole chromosome or chromosomal segment to yield whole chromosome or segmental aneuploidy, respectively. Aneuploidy has long been known to occur in *C. albicans* [7,19], including in many early laboratory isolates, such as CAI-4 [20] and WO-1 [21,22], as well as in many isolates taken directly from patients (reviewed in [7,23]) or isolated following passage through a mouse ([24]; A. Forche 2016, personal communication).

LOH is readily detectable in heterozygous diploid genomes as a contiguous homozygous genome region. There are three major types of LOH: short range, involving one or a few genes and thought to occur via gene conversion or double crossover events; chromosome arm LOH, thought to occur via a single crossover followed by co-segregation of homologous alleles or by break-induced replication and whole chromosome LOH, in which one homologue is lost, and the other is duplicated (either before or after the loss event, via re-replication or chromosome non-disjunction). Whole chromosome LOH is generally a downstream consequence of aneuploidy (either trisomy followed by chromosome loss or monosomy followed by gain of a copy of the remaining chromosome). Indeed, aneuploidy and LOH often occur together, as discussed further below.

This review focuses primarily on *C. albicans* and *C. neoformans*, a human commensal and an environmental saprophyte, respectively, that have become two of the most important human fungal pathogens worldwide in terms of incidence, morbidity and mortality. *C. albicans* is the most prevalent human fungal pathogen isolated from patients in hospitals in the developed world [25,26]. *Candida* infections are increasing in prevalence as a result of ever increasing numbers of profoundly immune-suppressed patients, either because of medical interventions such as cancer chemotherapy, solid organ or haematopoietic stem cell transplantations, as well as increased use of indwelling medical devices [27]. *C. neoformans* causes a meningoencephalitis in immunosuppressed patients and its incidence increased dramatically with the advent of the HIV epidemic since the 1980s, and currently is thought to cause over half a million deaths per year [28].

The role of aneuploidy is being increasingly understood as playing a role in both establishment of human infection and tolerance to antifungal drugs in fungal pathogens. These pathogens remain globally important, especially in the light of the persistence of the HIV epidemic and the growing prevalence of fungal infections in immunosuppressed patients. Importantly, ploidy change is one important mechanism by which these fungi adapt to environmental and host changes; it is likely that similar mechanisms provide adaptive responses in newly emerging fungal pathogens.

## 2. Ploidy change as a driver of increased diversity

A major take-home lesson from studies of *C. albicans* is that ploidy shifts are more frequent than previously assumed based on studies of model organisms under laboratory conditions. *C. albicans* was generally considered to be an ‘obligate diploid’. However, non-diploid isolates, including whole chromosome and segmental aneuploids, haploids, triploids, tetraploids and some with higher than tetraploid DNA content have been reported ([29–32] and reviewed in [7,23]).

The association of an acquired phenotype with a specific aneuploidy was first shown for monosomy of Chr5, which facilitates improved growth on sorbose [19,33]. This is thought to be owing to the presence of two or more unidentified genes on the right arm of Chr5 that inhibit the expression of the *SOU1* gene (located on Chr4). Other aneuploidies were found in isolates treated with antifungal drugs [34,35], with growth at elevated temperature [11] or with unconventional colony morphologies [36]. Aneuploidy arises through rare non-disjunction events, with up to 5% of drug-susceptible *C. albicans* isolates carrying at least one aneuploid chromosome or chromosome fragment [23,29–31,37]. In addition, some meiotic divisions yield aneuploidy: for example, up to 15% of the meiotic progeny of *Candida lusitaniae* are aneuploid [38]*.* Presumably aneuploidy is maintained in a strain if it provides a selective advantage under a given growth environment. This is not restricted to *C. albicans. S. cerevisiae* is used as a model in fungal evolution studies and can also cause infections in immunocompromised humans. Importantly, aneuploidy was recently reported to be a property of approximately one-third of *S. cerevisiae* clinical isolates. Whether these strains had been exposed to antifungal drugs or were drug-resistant was not reported [8]. Similarly, two of 19 *C. neoformans* clinical environmental isolates were aneuploid, although again, whether they had been exposed to antifungal drug or not is not specified [39].

Given that a characteristic of a given species is its stereotypical karyotype (number of chromosomes and chromosome organization), it follows that aneuploidy should be a transient state of altered chromosome copy number. Furthermore, it appears likely that within any large population a small number of cells will include aneuploid chromosomes owing to rare chromosome mis-segregation events. Most aneuploidies are likely to return to the basal ploidy level via chromosome mis-segregation and a fitness cost incurred by the aneuploid chromosome. However, under specific selective conditions, some specific aneuploid chromosomes appear to provide an advantage that promotes their selection. Furthermore, not all aneuploid chromosomes incur a high fitness cost and thus can be maintained for quite a while in the absence of selection.

One selective advantage that aneuploidy can provide in pathogenic fungi is drug resistance. A survey of clinical and laboratory isolates using comparative genome hybridization detected aneuploidy in half of all fluconazole-resistant isolates of *C. albicans* tested [40]. Most of these conferred resistance to other azole antifungals as well. Since that time, additional drug-resistant isolates have been analysed, and aneuploidy remains a prevalent property of many, although not all, drug-resistant or drug-tolerant isolates (e.g. [34,37,41], Feng Yang 2016, personal communication).

**Figure 1.**
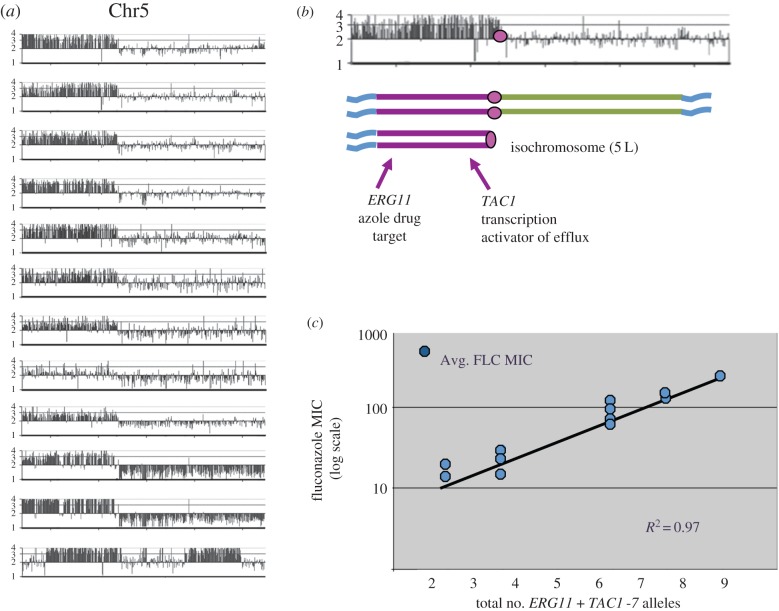
Some aneuploidies and LOH events enable drug resistance. (*a*) Isochromosome (5 L) was detected in 12 fluconazole-resistant isolates from a survey of 90 clinical and laboratory isolates. Shown is comparative genome analysis performed with in-house produced microarrays [40] with the data displayed along the eight *C. albicans* chromosomes as log_2_ ratios converted to ploidy levels (one, two, three or four gene copies) [40]. (*b*) Interpretation of i(5 L) geometry. *CEN5* (magenta circle) maps to the breakpoint where extra copies of Chr5 L begin. Chr5 L includes *ERG11,* which encodes lanosterol 14-α-demethylase, the target of azole antifungals and *TAC1,* which encodes a transcription factor that positively regulates expression of efflux pump genes (*CDR1* and *CDR2*)*.* (*c*) The number of copies of *ERG11 and TAC1-7* (a hyperactive allele of *TAC1* [43]) correlate well with the level of resistance (MIC) of the strain as determined by deletion analysis of isogenic strains derived from parent strain carrying isochromosome (5 L) [34]. FLC, fluconazole.

One specific aneuploidy, an isochromosome containing two extra copies of the left arm of Chr5 (isochromosome (5 L)), was associated with resistance to fluconazole in 20% of fluconazole-resistant isolates [19,40,42] (figure 1*a*). Two genes on Chr5 L (figure 1*b*,*c*) were responsible for the majority of the resistance of these strains: *ERG11*, which encodes the target of fluconazole, and *TAC1*, which encodes a transcriptional regulator of ABC-transporter drug efflux pumps Cdr1 and Cdr2 that reduce intracellular azole concentration. Thus, the acquisition of a single aneuploidy can confer resistance via two general strategies of drug resistance: increasing the levels of the drug target and decreasing the intracellular drug concentration. Furthermore, extra copies of these two genes were sufficient to reduce the extra resistance attributable to isochromosome (5 L) in a gene-dosage-dependent manner, supporting the idea that aneuploidy can confer drug resistance owing to the presence of additional copies of specific genes, rather than owing to the aneuploid state *per se* [34].

Similarly, in *C. neoformans*, the role of aneuploidy in phenotypic resistance to fluconazole is being increasingly observed. Under selective fluconazole pressure in a mouse model of cryptococcal meningitis, fluconazole-resistant colonies recovered from the mouse brain were found to be disomic for Chr1 [44].

Many other examples of new phenotypes caused by specific imbalances in specific genes on aneuploid chromosomes have been reported. For example, in *S. cerevisiae,* an extra copy of *AQY1* confers resistance to freeze–thaw cycles in environmental *S. cerevisiae* oak isolates [14], extra copies of *RLM1* increased the expression of cell wall components and facilitate the survival of cells lacking *MYO1*, which is considered essential for cytokinesis and cell separation [45]. Many more examples of aneuploidies that rescued the lethal phenotypes of deletions of essential genes were recently identified in a genome-wide screen [46]. Similarly, cells resistant to the Hsp90 inhibitor radicicol emerged from an extra copy of ChrXV, owing to elevated expression of two genes (*STI1* and *PDR5*) on that chromosome [47]; and resistance to 4-nitroquinoline-*N*-oxide (4NQO) is conferred by an extra copy of chromosome ChrXIII, and therefore an extra copy of *ATR1* [48,49]. Improved survival on caspofungin is seen in cells with Chr5 monosomy [50]. Thus, aneuploidy can confer drug resistance by increasing the copy number of genes involved in drug resistance or by changing the stoichiometry of gene products produced from the aneuploid chromosomes.

A major question in the field is whether ploidy state (diploid versus haploid) is sufficient to explain rates of adaptation. In a classic study, isogenic *S. cerevisiae* haploids and diploids were evolved in different concentrations of fluconazole [51], a drug that inhibits ergosterol biosynthesis. The timing of responses, as well as the mechanisms of resistance (altered ergosterol biosynthesis versus drug efflux) differed between haploids and diploids [51]. Theoretically, diploids contain increased numbers of target genes per cell, and thus should more rapidly acquire dominant mutations that exert a phenotypic effect with a single mutation than haploids. Yet despite this idea, haploids sometimes generated more mutants than diploids [52]. Consistent with this, *Candida glabrata*, a naturally haploid pathogenic yeast, is rising in prevalence, largely because of its higher levels of intrinsic drug resistance [53]. Yet, the most common resistance mechanism in haploid *C. glabrata* is activation of drug efflux via dominant mutations [54]. Thus, despite the idea that recessive mutations have a greater impact in haploids than in diploids, dominant mutations also play an important role in the acquisition of drug resistance by haploid as well as diploid pathogens.

With respect to ploidy, it is not clear that there is a simple rule that applies to all species, stresses and ploidy states. A ‘ploidy drive’ clearly brings most organisms back to their ‘base ploidy level’ [55]. Yet, *S. cerevisiae* tetraploids adapted to carbon limitation more rapidly than isogenic diploids and haploids; these tetraploids also acquired beneficial mutations with stronger fitness effects faster [56]. Analysis of many different stresses select for isogenic haploid or diploid *S. cerevisiae* isolates in different ways that clearly do not conform to a simple rule [57]. Much remains to be understood about the effect of ploidy on the degree of mutation. Nonetheless, it is clear that aneuploids and polyploids tend to undergo changes in chromosome number much more rapidly than do cells at their ‘base ploidy level’ [30,56].

## 3. Effects of ploidy shifts and aneuploidy on fitness

The role of ploidy and ploidy shifts in the emergence of pathogenic fungi is now beginning to be appreciated. The degree to which such ploidy changes drive the emergence of new fungal threats is not yet certain; however, it is clear that ploidy shifts and aneuploidy can promote the acquisition of altitude on the fitness landscape, even if the aneuploid state is transient. An example is *C. neoformans,* which has emerged from an environmental saprophyte to a pathogen of global importance [58]. Very large and polyploid (4n, 8n and 16n) cryptococcal cells, termed ‘titan cells’, were recently discovered and shown to be better able to tolerate oxidative and nitrosative stress [59], to prevent phagocytosis and contribute to dissemination to the central nervous system (CNS). In addition, the large size of titan cells protects them from phagocytosis by immune cells. Titan cells have been proposed to promote persistence in the host based on their prevalence in chronic lung infections [60]. Titan cells give rise to much smaller haploid or near-haploid cells, as well as diploids [61]. Interestingly, following exposure to fluconazole, a single titan mother cell can give rise to successive daughter cells that carry different aneuploid chromosomes [61], suggesting that titan cells promote the rapid production of diverse daughter cells. It appears, therefore, that aneuploid titan cells play a major role in the transition from environmental exposure to disseminated human infection. Similar adaptive chromosome-wide responses may contribute to the emergence of other emerging fungal threats.

One classical assumption of evolutionary theory is that when genetic changes confer increased fitness in a stress condition, the same changes are likely to incur a high fitness cost in the absence of the stress. Many aneuploids have been isolated from patients, suggesting that they were fit enough to compete successfully in the host (reviewed in [7,37]), yet they are less fit than laboratory strains when grown *in vitro*.

Despite classic evolutionary theories, aneuploidies that confer drug resistance do not necessarily have a high fitness cost in *C. albicans*. For example, isochromosome (5 L) confers a high fitness benefit in the presence of fluconazole (reviewed in [23]). Yet, in the absence of drug, strains carrying isochromosome (5 L) do not incur a large fitness cost. The isochromosome is not entirely stable and is lost at low frequency, especially following the heat shock treatment used for molecular transformations [34,62]. i(5 L) is not unique: there are a number of examples of resistant *C. albicans* strains that do not incur a fitness cost in the absence of drug [63–65]. Indeed, some isolates that are resistant to azoles owing to efflux pump activity are also more virulent, even in the absence of the drug [66,67].

Laboratory strains can also be aneuploid and, indeed, many early laboratory strains of *C. albicans* have one or more trisomic chromosomes without any obvious effect on fitness [20,32,68,69]. The consequences of these changes for different phenotypes continue to be discovered at the level of RNA and/or protein expression [70]. Studies performed with this strain and its derivatives need to take into consideration these LOH events: for example, the LOH on Chr3 in CAI-4 confers increased sensitivity to DNA damaging agents relative to the wild-type parent [71]*.* Thus, changes in ploidy affect laboratory as well as wild fungal isolates and these changes can cause broadly pleiotropic effects [72].

This ability to tolerate aneuploidy, and to maintain it in the absence of strong selection, was initially thought to be a quirky feature of *C. albicans,* especially because aneuploidies selected in a *S. cerevisiae* laboratory strain were highly unstable [73]. Yet aneuploidy is frequently detected in wild and clinical isolates of *S. cerevisiae* (reviewed in [74]), as well as in many other eukaryotic microbes including those responsible for emerging infections such as *Leishmania* spp. [75,76] and *Microsporidium* spp. [77]. Unstable aneuploidy was also found in chytrid isolates [78]. Additionally, it appears that aneuploidy may have a much stronger negative effect on the fitness of *S. cerevisiae* laboratory strains relative to wild isolates [17,49,73,79].

In addition, fitness may be fine-tuned to a specific host niche and/or may change with time in a given niche. For example, a *C. albicans* strain isolated from a blood stream infection (P94015) carried several complex segmental aneuploidies and exhibited an unusual morphology phenotype and gene expression pattern, drug resistance and surprisingly low virulence in a murine blood stream infection model [37]. Nonetheless, this strain exhibited better fitness when tested in a commensal mouse model of infection [37], consistent with it carrying a truncated copy of *EFG1,* which is involved in morphogenesis [80–84] and inhibits commensalism [69,85]. How is it that P94015 was isolated from the blood stream of a patient with a systemic infection, yet was only virulent in a commensal model? The authors proposed that the barriers to infection may have been very low in the immune-compromised host [37]. Alternatively, the isolate may have undergone microevolution in the host, consistent with the observation that successive bloodstream isolates from a chemotherapy patient exhibited reduced virulence over time in the patient [66]. In either case, measurements of virulence and fitness in the laboratory cannot recreate the situation *in vivo* perfectly*.*

A similar theme is evident for *C. neoformans,* where series of isolates from an infected individual were analysed following a relapsed infection [86]. In this case, the relapse isolate, F2, had two extra copies of the left arm of Chr12, grew faster at higher temperatures (37°C and 39°C), yet was unable to disseminate from infected lungs. The authors suggested that strain F2 underwent microevolution in the CNS, once dissemination was no longer required [86]. Again, we see that measurements of virulence and fitness may not always reflect the phenotypic adaptations of a specific isolate. Similarly, clinical strains of *C. neoformans* recovered from the CSF of an HIV patient were disomic for Chr13, and were correlated with reduced melanin production and consequently reduced virulence in mice [39]. The difference between growth conditions in the laboratory and in the wild (in patients) is likely to be different and is suggested to be the reason that many initially aneuploid *Saccharomyces* and *Candida* isolates often undergo changes in chromosome (Chr) copy numbers during propagation *in vitro* [7,16,78].

Reversion to the euploid state can mitigate the fitness costs incurred by aneuploidy and this often is detected when aneuploid cells are propagated in the absence of the environmental conditions that selected for the aneuploidy. Transient aneuploidy clearly has a critical role in the appearance of heteroresistance to fluconazole in *C. neoformans*, which also is associated with virulence [87]*.* As previously discussed, the appearance of resistance is associated with the disomy particularly of Chr1, and Chr 4 following exposure to the drug [5]. As well as being able to tolerate supra-MIC concentrations of fluconazole, the disomic clones were associated with virulence in a mouse model, with a significant increase in mortality in mice observed in those infected with strains with a higher frequency of disomy [87]. Importantly, when grown in the absence of drug selection, the aneuploid chromosomes are eventually lost, presumably as the disomy of Chr1 provided a selective advantage only when the drug was present.

*Cryptococcus gattii* is an emerging pathogen responsible for a major outbreak of cryptococcal meningitis in immune-competent patients. The *C. gattii* outbreak was first detected on Vancouver Island in 2004 and spread along the Pacific Northwest of the USA [88]. Interestingly, *C. gattii* also displays heteroresistance to fluconazole *in vitro,* although the role of aneuploidy as a driver for this has yet to be investigated.

Additionally, polyploid progeny of cryptococcal ‘titan’ cells, which as discussed above, play a key role in human pathogenesis, appear rapidly and are also transient [43,61]. Therefore, ploidy shifts in *C. neoformans* appear to be a transient phenomenon that can provide fitness costs or benefits in a specific host or niche [89].

Transient aneuploidy also may play an important role in other emerging fungal pathogens in the animal world. *Batrachochytrium dendrobatidis* (Bd) is a chytrid pathogen that is causing worldwide declines in frog populations. Isolates from infected frogs exhibited fewer aneuploidies following laboratory passaging for over 6 years [78]. This suggests that propagation *in vitro* exerted a selective pressure against aneuploidies found in earlier isolates. Thus, shift from the natural habitat to the laboratory appears to have altered chromosome copy number in many different fungi including Bd, with reductions in chromosome number associated with reduced virulence in laboratory studies [78].

There are little data regarding the role of aneuploidy in the spread of emerging plant pathogens. However, a variation on the theme, the presence of accessory chromosomes, is often seen in plant pathogens such as *Fusarium* spp. [6]. These accessory chromosomes appear to be critical for the host specificity of a given isolate [90]. However, isolates of the oomycete *Phytophthora infestans* [91], an emerging pathogen causing sudden oak death, recovered from the active site of infection in artificially inoculated oak trees, were found to display partial aneuploidy and LOH when compared with wild-type isolates. It remains unclear whether this aneuploidy incurs a fitness cost or if it provides a selective advantage in causing infection in the host.

Taken together, it appears that alterations in chromosome copy number are common events across the fungal kingdom in fungal pathogens, in laboratory isolates as well as in clinically important fungal pathogens. This highlights the idea that chromosome non-disjunction, which causes aneuploidy in a single cell division, occurs at relatively high frequency and has the potential to provide a fitness benefit that may facilitate adaptation to the stresses found within environments such as host niches. Nonetheless, it is a transient mechanism, as extra chromosomes can be lost and cells can return to the baseline karyotype.

## 4. Loss of heterozygosity as a long-term mechanism of rapid genome change

LOH is a common feature of *C. albicans* clinical isolates with a broad range of evolutionary diversity [37]. LOH events also can drive the acquisition of drug resistance [43,65]. Frequencies of LOH are much higher than the frequencies of point mutations (reviewed in [92]). In *C. albicans*, a specific LOH is detected at a frequency of approximately 10^−4^, and this frequency can increase 10- to 100-fold following exposure to stress [24]. The stress of growth *in vivo* also plays a role in altering LOH frequencies (A. Forche 2016, personal communication). All *C. albicans* isolates exhibit some degree of telomere-proximal LOH [37] as do many Bd isolates [78].

LOH events can become prevalent in a population over a relatively short timeframe. In series of clinical isolates from patients treated with an antifungal, novel driver point mutations acquired on a single homologue also undergo homozygosis in patients [93,94]. Another classic example is the ‘FH series’ of isolates from a single bone marrow transplant patient [95]. LOH of a hyperactive *TAC1* allele conferred increased resistance [43] in an early sample (FH3) and then persisted in the isolates with increased resistance levels (those that also carried i(5 L) [23,96]). It remains to be determined if the FH progenitor strain is highly prone to chromosome mis-segregation, recombination and/or mutagenesis or whether other genetic backgrounds would behave similarly under similar selective pressures. Sequencing of isolates from individual HIV patients treated by fluconazole [41] inferred driver mutations from the persistence of LOH events in consecutive isolates. In this case, three of the four of these isolates were more virulent than the progenitor strain from the same patient, when tested in the *C. elegans* model [97]. Recurrent recombination events have also been observed in *in vitro* evolution experiments*.* For example, in strain T118 evolved in increasing concentrations of fluconazole [98]*,* related recombination events generated i(5 L) in three different isolates [64]. Thus, LOH events are frequently associated with, and are often drivers of, phenotypic changes *in vivo* as well as *in vitro*.

Thus, both aneuploidy and LOH arise *in vivo* and these events can affect overall fitness, albeit in a gene-dependent, strain background-dependent and environment/niche-dependent manner. Specific aneuploidies and LOH events can improve fitness under specific environmental stress conditions.

Aneuploidies often appear coincident with an increase in drug resistance (as measured by MIC) and are more transient than LOH events [41,64,68], suggesting that the aneuploid state may facilitate the appearance of other mutations [99]. Elevated resistance levels that persisted in subsequent isolates were likely owing to additional LOH and/or small mutations that arose in response to the drug. This is consistent with the idea that aneuploidy may be a rapid solution enabling improved survival, which is subsequently replaced by one or more optimal solutions that incur lower fitness costs [100]. Of course, aneuploidy is transient, as whole chromosome aneuploidy is easily lost through a single chromosome non-disjunction event; by contrast, loss of an LOH requires acquisition of new information from a diverse partner via mating, a process that apparently occurs only rarely in pathogenic *S. cerevisiae* [101] and *C. albicans* [102].

## 5. Cell cycle processes that rapidly generate diversity: sex, parasex or no sex?

Knowing how emerging fungal pathogens generate diversity is critical for designing strategies to intervene in their spread. In most eukaryotes, sexual reproduction and recombination are among the major mechanisms for increasing genetic diversity [103]. Sexual life cycles require a series of ordered processes and do not ensue particularly rapidly. Interestingly, sexual reproduction appears to occur only infrequently in fungal pathogens of humans. Either some of the essential genes have been lost or only one of the two possible mating types is predominant in the environment [104]. Furthermore, it appears that isolates that adapt to the human host undergo far less recombination than do environmental isolates of the same species [101].

Several *Candida* species have only incomplete (parasexual) cycles, in which diploids of opposite mating type can mate to form tetraploids, and genome size reduction is achieved by random chromosome loss rather than meiosis (reviewed in [105,106]). The parasexual cycle has the potential to generate diversity through mitotic rather than through meiotic divisions [31,107]. Parasexual cycles have been observed primarily *in vitro* and just a few examples of *in vivo* mating have been reported [108,109]. Parasexual progeny acquire genetic diversity via chromosome loss, which is random and generates some degree of chromosome homozygosis as well as aneuploidy of one to three chromosomes. The chromosome loss process appears to follow random trajectories in which aneuploid intermediates can include many different combinations of homologues as well as different combinations of trisomic chromosomes [30], thereby producing diversity in a non-meiotic manner including rare, transient haploid intermediates [31]. How haploids form is not yet clear, although many haploids were isolated following passage in mice using a model of oropharyngeal candidiasis (Forche *et al*. 2016, unpublished data). The most parsimonious explanation would be that haploids form as a result of extensive chromosome loss. It remains unclear whether parasex occurs *in vivo* and whether parasex is the major mechanism by which aneuploid isolates are generated during growth within the human host.

## 6. Antifungals alter cell cycle progression to yield trimeras and aneuploids without parasexual mating

**Figure 2.**
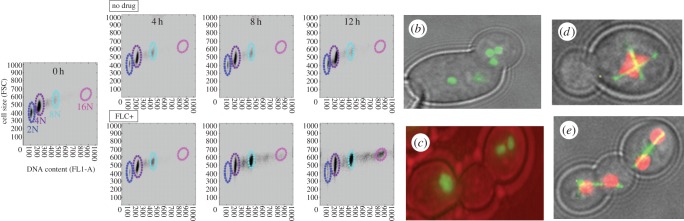
Antifungal drug exposure can induce aneuploidy and/or loss of heterozygosity. (*a*) Flow cytometry data comparing DNA content of Sytox green-stained cells with forward scatter levels as a proxy for cell size. Control diploids and tetraploid cells (derived by parasexual mating) were compared with samples without (top panels) or with (bottom panels) exposure to fluconazole for the indicated times. Note that DNA content and cell size increased in parallel in fluconazole-exposed cells. FSC, forward scatter. (*b*) A trimera cell after mitosis has four nuclei: the cell on the left contains two fused nuclei and subsequently underwent cytokinesis and cell separation to form a tetraploid; (*c*) example of multimera cells with multiple nucleoli detected using Nop1-GFP, green regions [110]; (*d,e*) time-lapse analysis of a cell that underwent unequal segregation of nucleoli (red) owing to failure of the two mitotic spindles (green) to remain parallel, resulting in very different amounts of nucleolar material being delivered to the two daughter cells (*e*) [110].

Because aneuploidy is so frequent in fluconazole-resistant *C. albicans,* we followed cell cycle dynamics in response to antifungal drug exposure. DNA content and cell cycle progression were monitored during the first hours of fluconazole exposure (figure 2*a*; [110]) and DNA content as well as cell size were measured by flow cytometry and increased with time in the drug (figure 2*a*). Fluorescence microscopy revealed two unconventional cell types: trimeras (figure 2*b*) and multimeras (figure 2*c*). Trimeras were evident within 4–8 h of drug exposure and continued to appear for at least 12–20 h after drug exposure. Multimeras, very large cells with unusually high numbers of nuclei/nucleoli (figure 2*c*), became evident after 12 h in drug. Trimeras and multimeras were alive and divided, albeit slowly [110].

**Figure 3.**
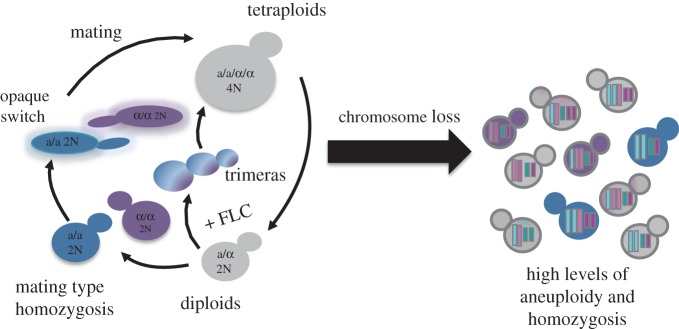
Model for mechanisms of ploidy shift in *C. albicans.* Diploids that become mating type homozygous and switch to the opaque state can undergo mating to form tetraploids. Alternatively, in the presence of fluconazole (+FLC), trimeras form and yield tetraploids. Tetraploids produced in either way can undergo chromosome mis-segregation and a reduction in ploidy to return to a near-diploid state, which can include chromosomes that underwent a gain or loss and/or are aneuploid. This generates genotypic and phenotypic diversity without meiotic divisions [29,30]. Trimeras appeared after 4–24 h; mutlimeras were much less prevalent, but at least a few multimeras were evident by microscopy.

Cells exposed to drug undergo a series of stereotypical events to become aneuploid via a tetraploid intermediate (figure 3). This involves changes in the regulation of cell cycle progression (nuclear/spindle division is uncoupled from cell growth) and a failure of cytokinesis (resulting in mother and daughter cells sharing a contiguous cytoplasm). The next dramatic defect was the production of a single new bud, to form a trimera—a set of three continuous cell ‘compartments’ (grandmother, mother and daughter cells). A fourth bud failed to form, perhaps owing to limiting membrane components or inhibition of multiple budding events in the contiguous cytoplasm [110]. This resulted in three cell compartments and two nuclei that undergo mitosis to yield four nuclei—a problematic situation in which one of the daughter compartments necessarily receives two nuclei—either via collapse of a mother–daughter pair to reform a single nucleus or via formation of a dikaryotic cell composed of two unfused nuclei.

The resulting tetraploid or dikaryotic nuclei contain two spindles, a situation that clearly drives chromosome mis-segregation (figure 2*d*). This multi-spindle state presumably occurs owing to a failure to fuse the spindle pole bodies, as a process that occurs during conjugation, for example in *S. cerevisiae* [78]. What is not clear is whether parasexual mating in *C. albicans* also results in more than one spindle. Ultimately, aneuploid cells continue to undergo chromosome mis-segregation and stabilize in a near-euploid state that is usually, but not always near-diploid [30].

Importantly, trimera progeny are at least as viable as non-trimera progeny. They (as well as multimeras) yielded viable colonies; the colonies were often aneuploid and were able to survive on drug at least as well as non-trimera cells. Of note, trimeras form *in vivo* as well, soon after fluconazole is administered in a model that enables visualization of fungal cells growing within the host mouse ear [111]. Furthermore, different azole antifungals as well as caspofungin can induce trimera formation. Trimera formation in azole antifungals was evident in other non-*albicans Candida* species [110] and appears to be occurring in *C. neoformans,* a basidiomycete pathogen, as well (Lukasz Kozubowski 2016, unpublished result). Thus, formation of aneuploids via a tetraploid intermediate may turn out to be a common mechanism in eukaryotes, ranging from cancer cells [112] to ascomycete and basidiomycete fungi. Furthermore, it appears to be a common response to high levels of stress such as drug exposure.

If a specific chromosome imbalance confers a selective advantage in a given stress (e.g. a given antifungal drug), cells containing that specific aneuploid chromosome constellation should become enriched in a population (e.g. in the presence of the drug). If the selection coefficient is large, this could happen very quickly. We propose that events of this sort would promote the rapid appearance of new isolates with strong selective advantages over their progenitors and could help explain the emergence of new fungal pathogens. Accordingly, we propose that the series of events through which mitotic collapse leads to tetraploidy, aneuploidy and then near-euploidy, provides a mechanism of generating highly diverse genomes that yield some progeny better adapted to a specific selection pressure. This process appears to be quite general, as analogous mechanisms involving polyploidization followed by chromosome mis-segregation clearly operate in cancer cells [94] and aneuploidy appears to be well tolerated across the fungal kingdom [113].

In summary, it appears that in fungi, and specifically in human pathogenic fungi such as *C. neoformans* and *C. albicans*, survival of stress may not require the fastest growth rate, nor the frequent operation of a sexual or parasexual cycle. Rather, mitotic defects have the potential to produce trimeras, tetraploids and aneuploid progeny rapidly. These diverse progeny have the potential to survive and evolve under the selective pressure of acute stress conditions.

The series of events that lead to a specific adaptive outcome may differ between cells, but likely involve aneuploidy, LOH and the acquisition of smaller-scale changes at the nucleotide level. As new fungal threats continue to emerge, consideration of these adaptive mechanisms may help us to understand the genetic basis for their new found success. The genomic diversity afforded by whole scale changes in chromosome copy number may be far greater than currently appreciated, and even transient changes, in the right place and right time, may contribute to the emergence of previously non-pathogenic fungi as threats to plants, animals and humans alike. Ultimately, the ability to survive and adapt is critical to the emergence of new pathogens, and the mechanisms here provide rapid, if not elegant, first steps toward adaptation to new hosts and environments. This is consistent with the often-paraphrased Darwinian adage: …it is not the strongest of the species that survives; but the ones best able to adapt and adjust to the changing environment … [114, p. 4].

## Data accessibility

No primary data are presented other than the figures already present within the manuscript. There is no electronic supplementary material.
